# Aortopulmonary collateral artery in prenatal exposure to carbamazepine – endovascular therapy and technical considerations: a case report

**DOI:** 10.1186/s13256-015-0645-1

**Published:** 2015-08-27

**Authors:** Gloria Pelizzo, Valeria Calcaterra, Savina Mannarino, Lorenzo Paolo Moramarco, Giovanni Leati, Pietro Quaretti

**Affiliations:** 1 Department of the Mother and Child Health, Pediatric Surgery Unit, IRCCS Policlinico San Matteo Foundation and University of Pavia, P.le Golgi n.2, 27100 Pavia, Italy; 2 Department of the Mother and Child Health, Pediatric Unit, IRCCS Policlinico San Matteo Foundation, Pavia, Italy; 3 Department of Internal Medicine, University of Pavia, Pavia, Italy; 4 Unit of Interventional Radiology, IRCCS Policlinico San Matteo Foundation Pavia, Pavia, Italy

**Keywords:** Aortopulmonary collateral, Plug occlusion, Carbamazepine, Children, Multidisciplinary

## Abstract

**Introduction:**

Aortopulmonary collateral arteries are an uncommon variant of alternative blood supply in cases of complex congenital heart disease. Although surgery may still be the classic approach for this condition, mini-invasive endovascular occlusion has been recently attempted as an alternative less traumatic procedure. Children born to women with epilepsy are at increased risk of congenital malformations.

**Case presentation:**

A cardiovascular malformation in a 6-year-old white boy with prenatal exposure to carbamazepine is described. At birth, he underwent atrial-ventricular septal defects repair. At 6 years of age, he was diagnosed to have an aberrant aortopulmonary artery from the descending aorta. He presented with recurrent respiratory infections and no cardiovascular signs, but there was associated right upper lobe hyperperfusion. Collateral percutaneous plug embolization was performed because of risk for cardiorespiratory infections, pulmonary hypertension and atrioventricular dilatation. The post-releasing control showed a complete occlusion of the aberrant artery. A chest radiogram and computed tomography showed normalization of vascular pattern of his right lung at 9-months follow-up. No complications and no respiratory infections in the first follow-up year were observed. A good growth gain was obtained.

**Conclusions:**

Plug embolization in an aortopulmonary collateral artery is an interesting alternative to surgery and is suitable for children with minor congenital heart disease and without severe respiratory and/or cardiovascular symptoms. Management and long-term pediatric multidisciplinary follow-up is recommended. Prenatal exposure to carbamazepine could be considered in the pathogenesis and diagnosis of the malformation.

## Introduction

Major aortopulmonary collateral arteries are reported in patients with cyanotic congenital heart disease and reduced pulmonary blood flow such as tetralogy of Fallot (TOF). These collaterals are also noted in neonates, especially premature newborns with bronchopulmonary disorder [1, 2].

The aberrant artery arises from the aorta and supplies one or more pulmonary regions. The lung receives systemic arterial blood from the aorta resulting in lung hyperperfusion and increased pulmonary blood pressure.

Surgery is the conventional treatment for this condition; however, percutaneous vascular occlusion has recently been attempted as a mini-invasive procedure [3, 4].

Approximately 1% of all pregnancies are in woman with epilepsy. Antiepileptic drugs (AEDs) have the potential to affect development throughout pregnancy [5–8]. Children born to women with epilepsy are at increased risk of malformations, such as heart, orofacial, urologic, skeletal and neural tube defects.

We describe a “pseudo” isolated aortopulmonary collateral artery in a child with prenatal exposure to carbamazepine. Indications to endovascular treatment with plug embolization and technical considerations are discussed.

## Case presentation

A 6-year-old white boy was diagnosed to have a large aortopulmonary collateral artery during preoperative evaluation for adenoidectomy. He had a history of recurrent respiratory tract infection, symptoms of gastroesophageal reflux and scoliosis. Saturation was normal and no abnormal auscultation findings were noted. His weight was 20.7kg (25–50 percentile) and height 119cm (75 percentile).

Prenatal exposure to carbamazepine because of maternal epilepsy was reported. He was delivered at term (birth weight 3100g). He underwent ventricular and atrial septal defects correction in the perinatal period. Since birth facial malformations (long philtrum, micrognathia, epicanthic fold) and paralysis of facial muscles resulting from dysfunction of the seventh cranial nerve were documented. DiGeorge syndrome and array-CGH deletions were excluded.

**Fig. 1:**
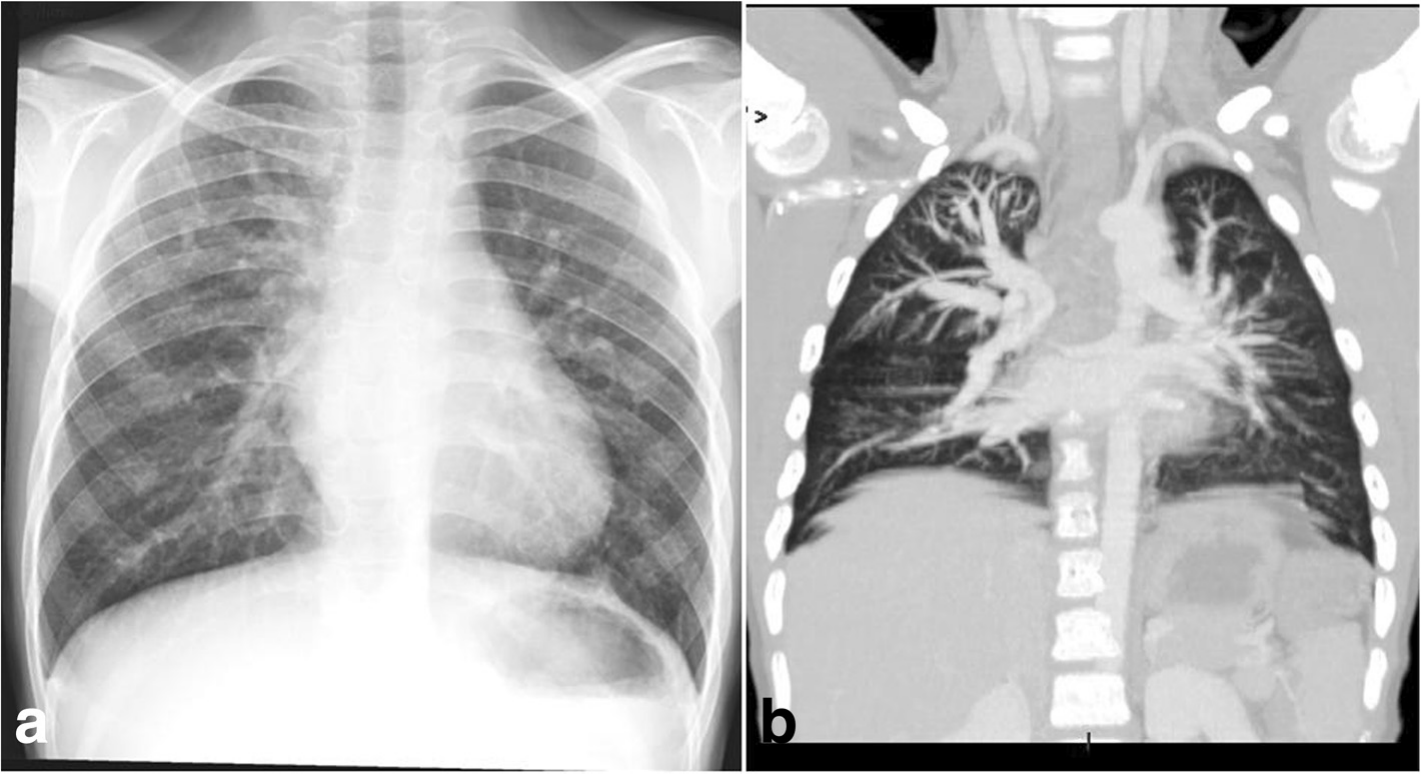
Pre-procedure chest radiogram (Panel **a**) and coronal multiplanar reconstruction (Panel **b**) of contrast-enhanced multidetector computed tomography showing increased pulmonary vascularity of the right lung mainly in the upper lobe

**Fig. 2:**
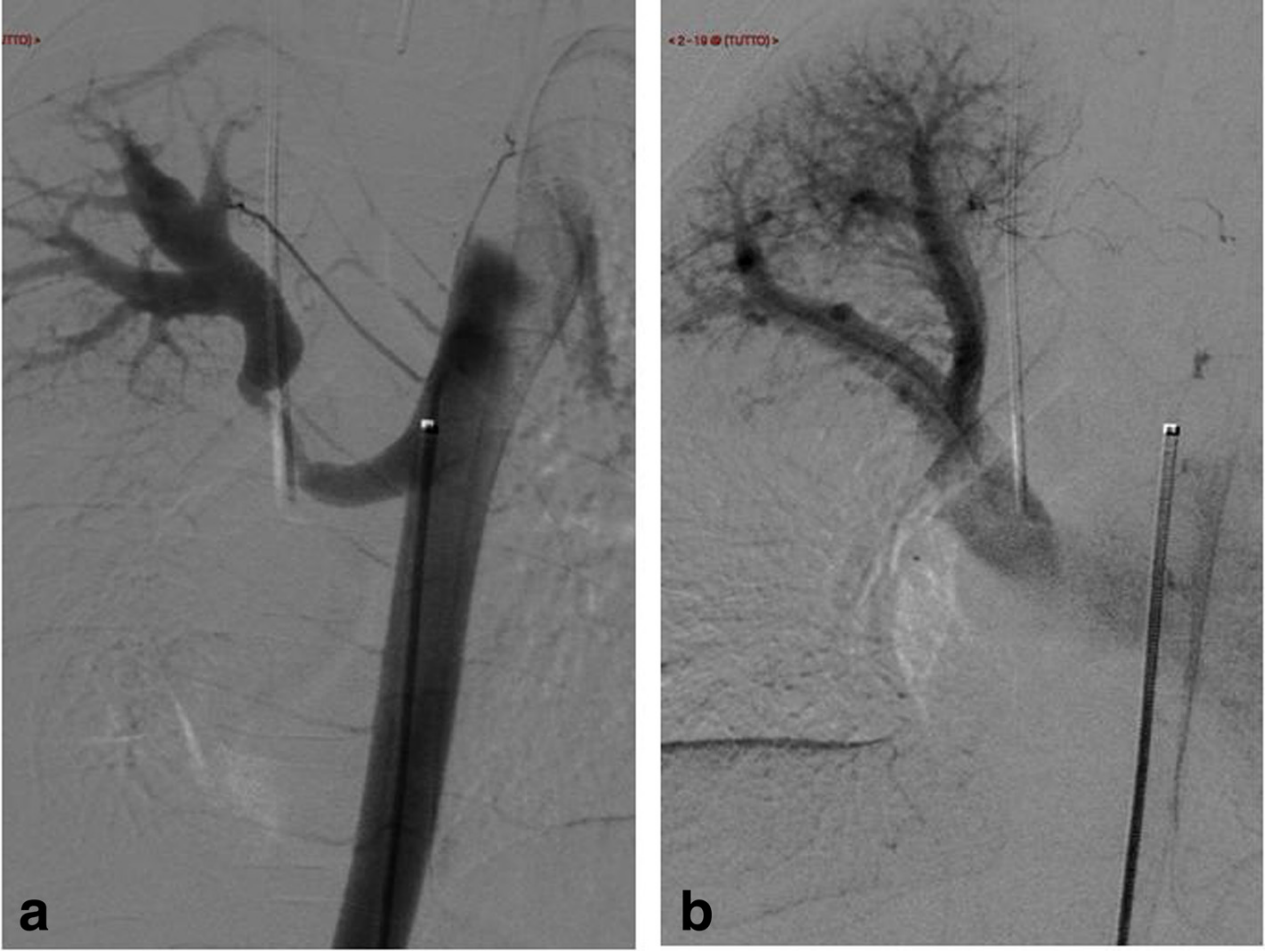
Aortogram showing aortopulmonary collateral artery opacifying the lobar superior pulmonary artery (Panel **a**). Hyperperfusion of the right upper lobe and hypertrophied venous return (Panel **b**)

**Fig. 3:**
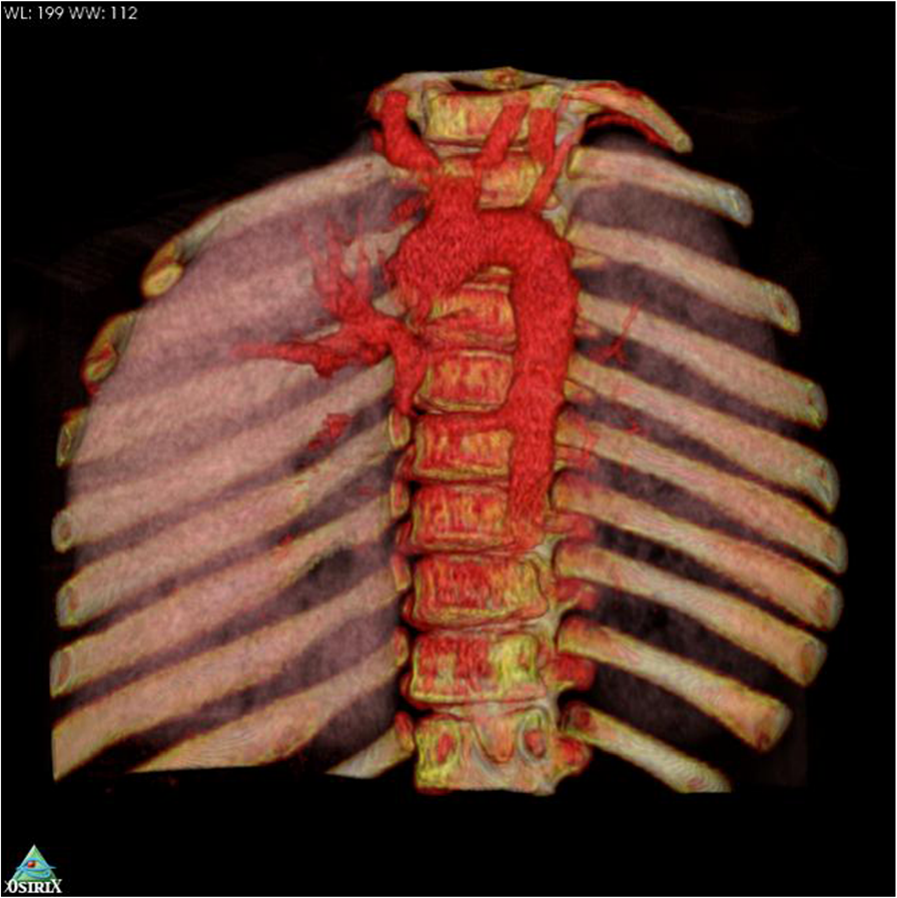
Three-dimensional volume rendering of the malformation

On chest X-ray were signs of bronchitis and hyperperfusion of his right lung (Fig. 1). An echocardiogram showed no shunts and no evidence of ventricular overload. A computed tomography (CT) angiography scan revealed an abnormal vessel arising from the thoracic descending aorta coursing toward his right upper lung. On endovascular occlusion, the angiogram confirmed the hyperperfusion of the right upper lobe (Fig. 2). The diameter of the aberrant vessel was up to 8mm. No lung anomaly or other abnormal vessels were detected. Three-dimensional volume rendering of the malformation is shown in Fig. 3.

Pulmonary hyperperfusion is a risk factor for recurrent cardiorespiratory infection and lung hypertension. This risk is probably higher in syndromic malformations. A mini-invasive vascular occlusion was attempted after a multidisciplinary evaluation (pediatric cardiologist, pediatrician, pediatric surgeon and interventional radiologist).

**Fig. 4:**
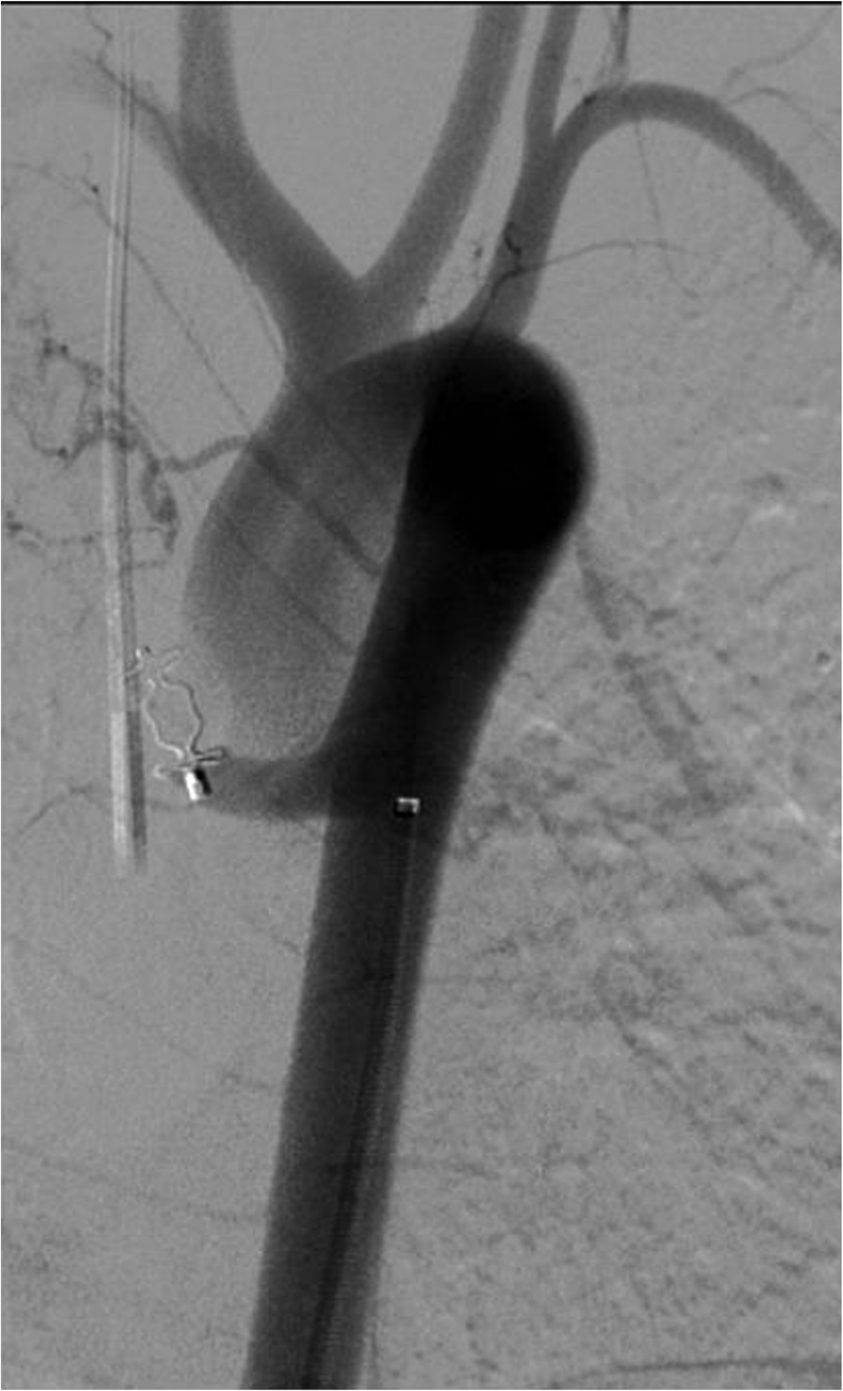
Plug release and closure of the aortopulmonary collateral artery. Five minutes aortogram control

Under general anesthesia, ultrasound-guided right femoral common artery access was gained. The major aortopulmonary collateral artery (MAPCA) was easily catheterized with a 4F vertebral Glidecath catheter. The hypertrophied pulmonary arteries of the superior lobe were opacified as well as the normal venous drainage excluding a lobar sequestration. Due to the very short length and ascending direction of MAPCA, coils as embolic agents were excluded because of the high risk of nontarget embolization. Through the Glidecath a 6mm bi-segmentary Amplatzer plug type IV was released but it appeared undersized and unstable. The plug was withdrawn from the outer portion. The Glidecath 4F was exchanged over a stiff wire with a 4F 55cm-long Cook sheath with its distal tip in the pulmonary tree. A new tree segments (or lobes) 8mm Amplatzer plug type II was released through the sheath inside the MAPCA. At completion angiography through the side arm of the sheath the MAPCA was embolized (Fig. 4). Hemostasis was done at the femoral entry by manual compression.

**Fig. 5:**
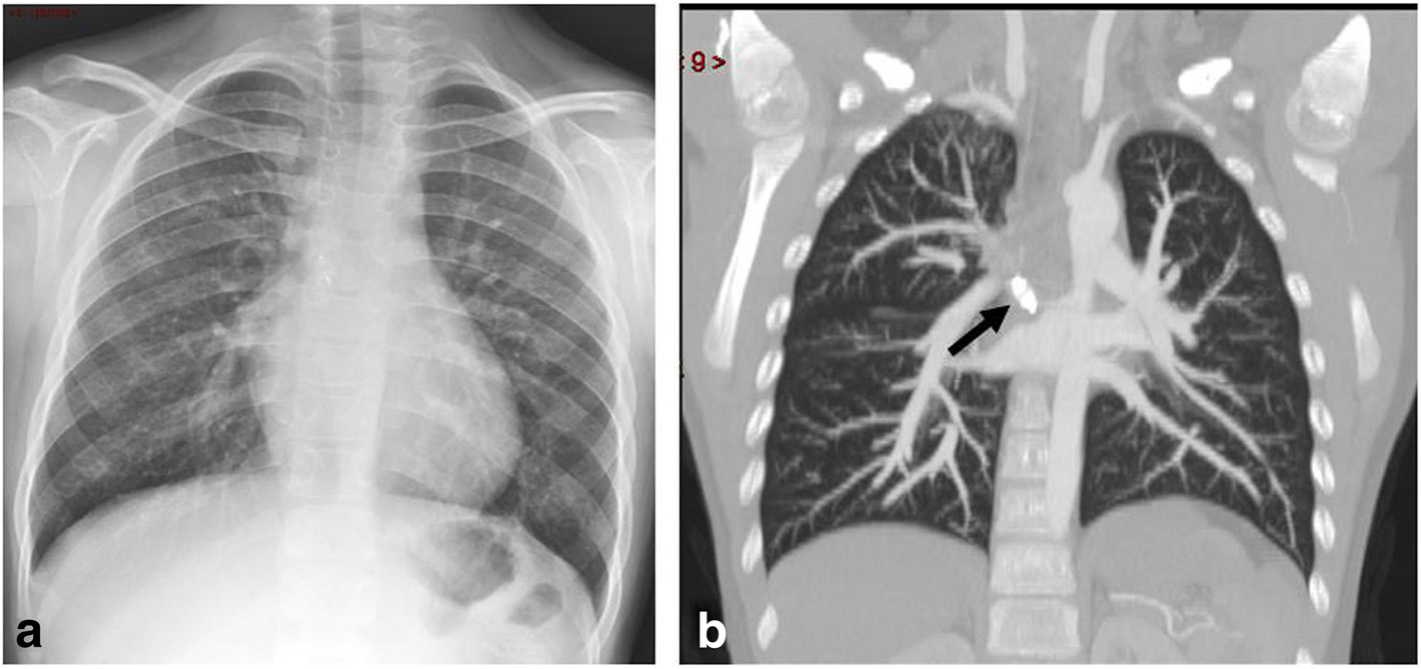
Chest radiogram (Panel **a**) and coronal multiplanar reconstruction (Panel **b**) of contrast-enhanced multidetector computed tomography showing normalization of the vascular pattern of the right lung (at 9-months follow-up). Vascular plug (*arrow*)

A chest radiogram and CT showed normalization of the vascular pattern of the right lung at 9-months follow-up (Fig. 5).

No complications and no respiratory infections in the first follow-up year were observed. A good growth gain was observed: weight 25kg, 75 percentile; height 121cm, 75 percentile.

## Discussion

Aortopulmonary collateral arteries are usually associated malformations in patients with severe congenital cardiac disease and reduced pulmonary blood flow such as TOF. Neonatal pulmonary injury, hypoxia, trauma or inflammation could act as stimuli for the persistence of the collaterals in postnatal life [2]. Systemic arterial supply to normal lung parenchyma with regular bronchial tree, without any congenital malformations is a rare disorder [1, 2].

The diagnosis of aortopulmonary collateral arteries in carbamazepine syndrome with non-severe congenital heart malformations, as ventricular and atrial septal defects has not been described yet.

Carbamazepine is one of the most commonly used AEDs in Europe among women of childbearing age. Even though the vast majority of infants exposed to AEDs *in utero* are born healthy, AEDs remain one of the most common potentially teratogenic agents in pregnancy. A correlation has been reported in several studies between carbamazepine and both minor (including the term “fetal carbamazepine face” described as epicanthic folds, short nose, long philtrum, upward slanting palpebral fissure, nail hypoplasia, front bossing, malar hypoplasia and micrognathia) and major abnormalities (cardiac malformations, neural tube defects, urinary tract, skeletal abnormalities, and cleft lip/palate) [5–8]. The exposure to AEDs during pregnancy could play a role in the pathogenesis of aortopulmonary malformations.

The aberrant vessel commonly arises from the descending thoracic aorta, from the proximal abdominal aorta or the celiac artery. Venous drainage is always via the normal pulmonary vein. CT angiography is a useful technique for differential diagnosis. Patients could have no symptoms for several years after birth. In adult age, hemoptysis and acute chest pain caused by pulmonary hypertension may be present. In children it may be discovered following the incidental finding of a continuous chest murmur. The persistence of lung overperfusion may predispose to respiratory infections, as in our case.

In the past, surgery was the elective approach to ligate an abnormal vessel. However, in recent years, the endovascular approach has replaced surgical treatment. A variety of techniques/devices are available for percutaneous vascular occlusion. Plug embolization is a safe, fast and precise technique for the occlusion of large vessels in young patients. Previous studies have described the feasibility of this technique in children with TOF, veno-venous or arteriovenous collaterals in functional univentricular hearts, pulmonary atresia with a ventricular septal defect, large coronary artery fistula to the right atrium and in preterm [1, 4]. There were no studies of interventional treatments using plug embolization of an anomalous systemic artery in children with normal lung and non-severe congenital heart disease.

There have been reports of the use of coils to embolize MAPCA. The more frequent side of MAPCA is toward the left lobar inferior pulmonary arteries where its length and course allow the artery to be safely filled with coils. In our case coiling was ruled out due to the straight and short course of MAPCA with high risk of migration and nontarget embolization both distally in pulmonary arteries than proximally in aorta branches. The choice of plug was obliged. The use of a 55cm-long 4F introducer allowed us to release a larger and safer tree segments 8mm Amplatzer plug type II. Usually the Amplatzer plug type II requires a 6F guiding catheter to be released; the choice of a long introducer enabled us to maintain a 4F-diameter approach without enlarging the femoral entry, which would potentially reduce hemostasis complications. MAPCAs up to 8 to 16mm of diameter can be occluded through 4 to 6F sheaths by means of Amplatzer plug type II.

## Conclusions

Plug embolization in an aortopulmonary collateral artery is an interesting alternative to surgery and is suitable for children with minor congenital heart disease and without severe respiratory and/or cardiovascular symptoms. A lung segment with overperfusion may be vulnerable to respiratory infection and pulmonary hypertension. A minimally invasive approach is the treatment of choice, if it is feasible and safe. Pediatric multidisciplinary management and follow-up is recommended. Prenatal exposure to carbamazepine could be considered in the pathogenesis and diagnosis of the malformation.

## Consent

Written informed consent was obtained from the patient’s legal guardians for publication of this case report and any accompanying images. A copy of the written consent is available for review by the Editor-in-Chief of this journal.

## Abbreviations

AEDs: Antiepileptic drugs
CT: Computed tomography
MAPCA: Major aortopulmonary collateral artery
TOF: Tetralogy of Fallot
